# Non-Occlusive Mesenteric Ischemia Due to Hirudotherapy: A Case Report

**DOI:** 10.7759/cureus.9467

**Published:** 2020-07-30

**Authors:** Çağrı Akalın, Nergis Ekmen

**Affiliations:** 1 Department of General Surgery, Ordu University Training and Research Hospital, Ordu, TUR; 2 Department of Gastroenterology, Gazi University Hospital, Ankara, TUR

**Keywords:** leech therapy, acute mesenteric ischemia, diabetic foot

## Abstract

Leech therapy, which can lead to complications such as anemia and bleeding, has been used to treat many diseases since ancient times. Furthermore, some substances in leech saliva are known to have anticoagulant effects. Acute mesenteric ischemia, which develops due to mesenteric vascular obstruction, can be treated medically or surgically. Non-occlusive mesenteric ischemia (NOMI) occurs as a result of decreased blood flow in mesenteric vessels due to hypovolemia, hypotension, etc. In this report, we mentioned a 57-year-old male patient who was admitted to the emergency department with syncope and weakness. In his medical history, the patient was stated to have used leech therapy to treat diabetic wounds on his feet, and prolonged and unstoppable bleeding was seen after leech bites. On his physical examination, there was tenderness in all quadrants of the abdomen. Abdominal computed tomography without contrast agent showed hepatic portal venous gas and pneumatosis cystoides intestinalis (PSI). The patient underwent laparotomy owing to the development of acute abdomen during the follow-up. Necrosis was seen in the terminal ileum and entire colon. Low flow in mesenteric vascular vessels of these necrotic segments was indicated with intraoperative Doppler ultrasonography. All necrotic segments were resected and open end-ileostomy was performed. The patient was discharged on the 17th day of follow-up. In conclusion, excessive bleeding caused by leech therapy can cause NOMI.

## Introduction

Leech therapy has been used in the treatment of some diseases since ancient times in the medical field. This treatment is known as hirudotherapy and is used for many diseases such as cardiovascular disease, diabetes mellitus and its complications, after replantation treatment, revascularization, and soft tissue injury [1]. Bleeding and anemia are among complications of hirudotherapy [2]. Vascular obstruction of the intestine causes acute mesenteric ischemia (AMI). AMI has a high prevalence and rates of mortality (50%-69%) in patients with cardiovascular disease and over 50 years old [3].

In this paper, the aim was to report a case of AMI which developed after acute bleeding and anemia leech therapy of a diabetic foot ulcer.

## Case presentation

A 57-year-old man was admitted to the ED with complaints of syncope and weakness. We noticed that in his medical history, leeches were applied to treat the diabetic wounds on his feet two hours before he attended our clinic. After this, the leeches spontaneously detached but he observed bleeding from his bites and bleeding did not stop in spite of compression with tight bandages. A review of the patient’s past medical history revealed hypertension, diabetes mellitus, diabetic foot, ischemic heart disease and coronary artery bypass surgery.

**Figure 1:**
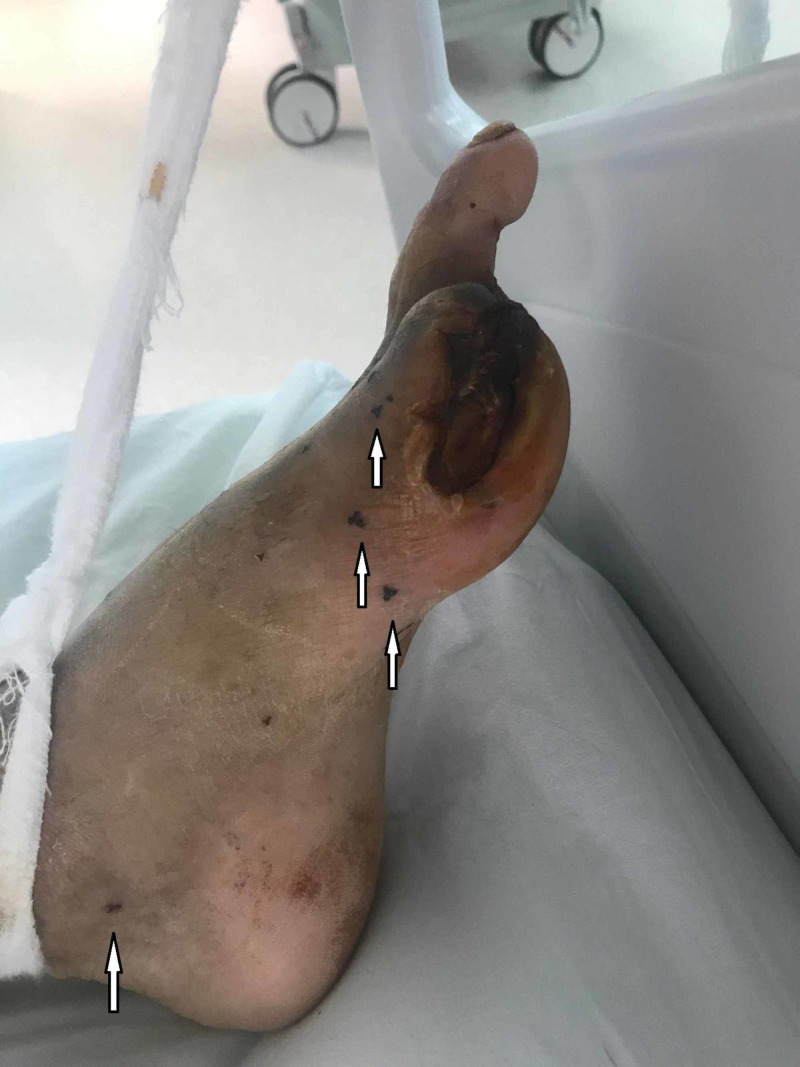
Diabetic foot ulcer under the first metatarsal head of his left foot and multiple leech bites (white arrows)

Vital signs were as follows; fever: 36.6°C, blood pressure: 85/50 mmHg, heart rate: 112/min and respiratory rate: 28/min. Physical examination revealed tenderness in the whole abdomen. Ampulla was empty with rectal examination. There was an ulcerative lesion with size 5x3 cm on the site of the amputated first toe. Additionally, there were multiple bites due to leech therapy on both feet (Figure 1).

**Figure 2:**
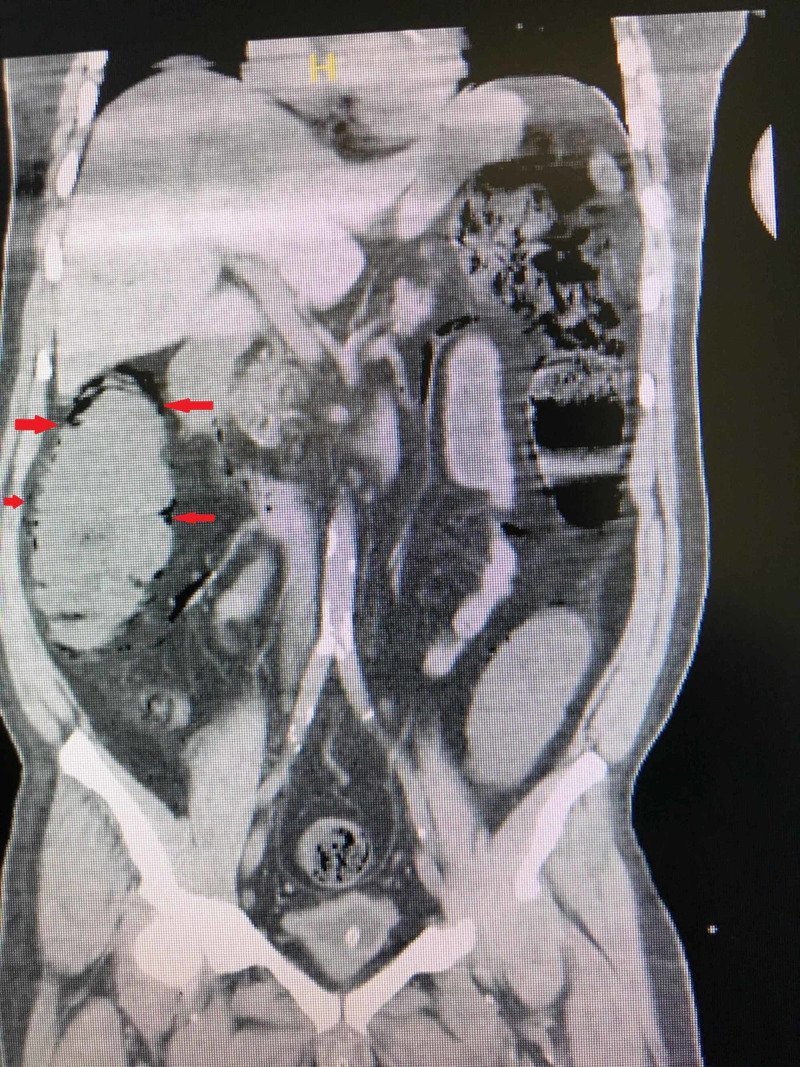
CT scan, coronal plane Red arrows are showing the pneumatosis cystoides intestinalis in the bowel wall

**Figure 3:**
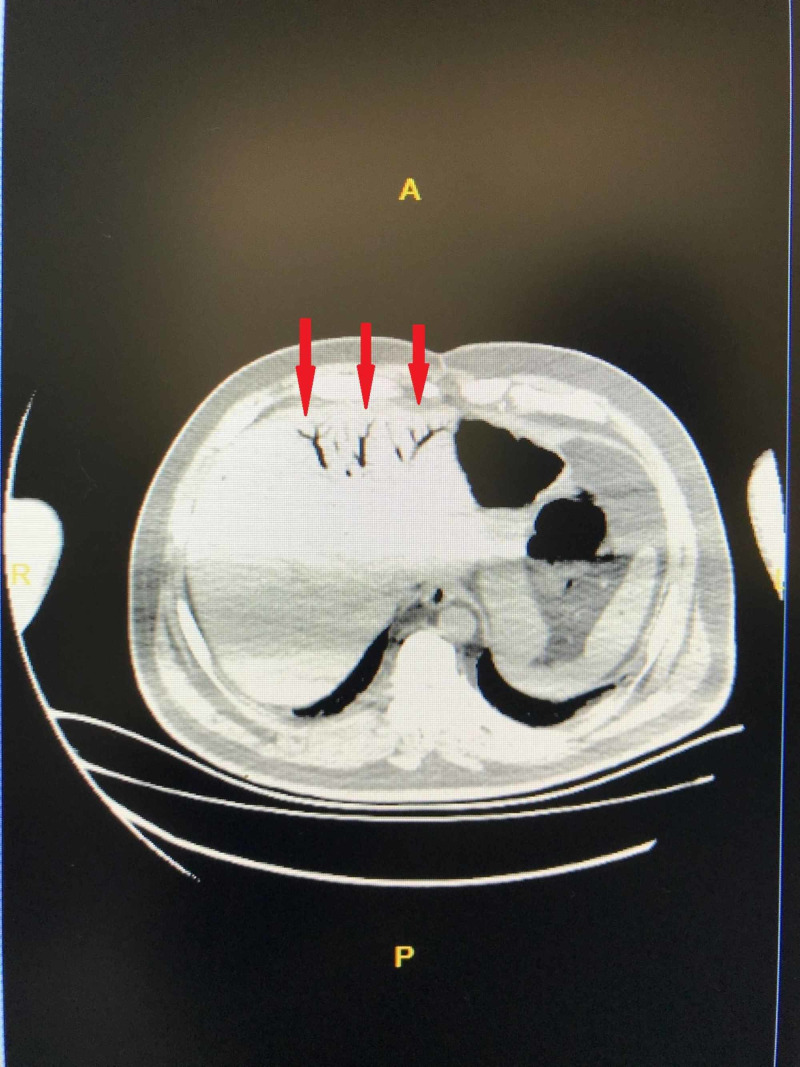
CT scan, axial plane Red arrows are showing the hepatic portal venous gas in the center and left  lobe of liver

Laboratory tests indicated the following: hemoglobin: 7.6 g/dL, white blood cells: 9370/mm3, platelets: 147000/mL, blood urea nitrogen: 29.1 mg/dL, creatinine: 1.98 mg/dL, glucose: 527 mg/dL, Na: 127 mmol/L, K: 6 mmol/L, CL: 94.5 mmol/L, C-reactive protein: 1.27 mg/dL and other routine blood tests and coagulation functions were all normal. Blood gas analysis revealed pH: 7.26, pO2: 86 mmHg, and bicarbonate: 20.2 mmol/L. Abdominal ultrasonography (USG) showed gas in the intrahepatic biliary ducts. No obstructive pathology was found to mesenteric blood flow on Doppler USG. CT without intravenous contrast was performed because of abnormal renal function tests. CT revealed gas near the stomach, among colon segments (pneumatosis cystoides intestinalis, PSI) and intrahepatic biliary ducts in central and left hepatic lobe (hepatic portal venous gas, HPVG) (Figure 2, Figure 3, respectively).

The patient was referred to another hospital that had an intensive care unit and started conservative treatment with antibiotics and intravenous fluid. Twelve hours after treatment, his general condition did not improve and signs of diffused peritonitis were observed on abdominal examination. The patient underwent exploratory laparotomy. Necrosis was found in the terminal ileum and the entire colon during laparotomy. Doppler USG detected low flow in the vessels supplying these necrotic segments. However, resection was decided due to necrosis of these segments. These necrotic intestinal segments were resected and an end-ileostomy was performed. He had an uneventful recovery and was discharged on the 17th postoperative day.

## Discussion

Hirudotherapy is known as a complementary treatment method applied with blood-sucking leeches. Leeches are attached to the skin of the diseased area and the aim is to acquire potential utility from leech saliva that is secreted while the leeches are feeding. It has been used for centuries and is still used in Asian and European countries [4,5]. The saliva of the leech contains hirudin, histamine-like substances, hyaluronidase and collagenase. Hirudin has only a transient antithrombin effect in humans and is a powerful anticoagulant. While leech bites, hyaluronidase and collagenase allow it to reach the tissues and blood vessels; histamine-like molecules cause vasodilatation, and platelet functions, kinin activity, and the coagulation cascade are inhibited [6,7]. We think that hypovolemia due to prolonged bleeding caused syncope in this case.

AMI occurs as a result of decreased blood flow to the intestines. AMI is classified into four groups according to its etiology: arterial embolism of the superior mesenteric artery (SMA), arterial thrombosis of SMA in a pre-existing atherosclerotic lesion, mesenteric venous thrombosis, and non-occlusive mesenteric ischemia (NOMI). NOMI is responsible for 20%-30% of AMI episodes. Especially in the presence of atherosclerotic disease, mesenteric ischemia occurs due to mesenteric vasospasm without arterial or venous obstruction [8]. We think this theory formed the etiology in this case. Besides, mesenteric vasospasm without arterial or venous obstruction is known. This knowledge helps us to understand the development of NOMI in a hemodynamically-stabilized patient after treatment [9]. Doppler USG is insufficient to show distal embolism of main vessels and for the diagnosis of NOMI. Angiography is the gold standard for diagnosis of mesenteric ischemia. Angiography is the most reliable method to show the presence and localization of occlusion [10]. In this case, we could not perform mesenteric angiography because of abnormal renal function tests. We did not find the exact reason for AMI. However, the detection of blood flow in necrotic segments supports the diagnosis of NOMI.

HPVG is thought to be caused by bacterial gas formation from an abscess in the intestinal lumen or liver and bacteria that cause gas formation in the portal vein [11,12]. The mortality rate in HPVG is between 75% and 90% and is higher in patients associated with necrotizing enterocolitis and mesenteric vascular occlusions [13]. PSI is characterized by intramural gas-filled cystic formations involving any part of the gastrointestinal system. Most of the time the disease involves the jejunum and ileum; moreover, the colon is affected in 6% [14]. The findings of AMI on CT are gas in the portal vein, edema of the intestinal wall, mesenteric edema and PSI [15]. Also, in our case, PSI and HPVG were detected on CT. Peritoneal irritation signs were found during the physical examination and after this diagnosis was established with emergency laparotomy.

## Conclusions

In summary, hirudotherapy may induce NOMI due to prolonged bleeding. Leech therapy should be performed by certified experts in appropriate conditions. Patients should be closely monitored for its complications.
